# Accelerated high frequency rTMS induces time-dependent dopaminergic alterations: a DaTSCAN brain imaging study in healthy beagle dogs

**DOI:** 10.3389/fvets.2023.1154596

**Published:** 2023-05-16

**Authors:** Yangfeng Xu, Kathelijne Peremans, Sofie Salden, Kurt Audenaert, Andre Dobbeleir, Ann Van Eeckhaut, Dimitri De Bundel, Jimmy H. Saunders, Chris Baeken

**Affiliations:** ^1^Department of Head and Skin, Ghent Experimental Psychiatry (GHEP) Lab, Ghent University, Ghent, Belgium; ^2^Department of Morphology, Imaging, Orthopedics, Rehabilitation and Nutrition, Faculty of Veterinary Medicine, Ghent University, Merelbeke, Belgium; ^3^Department of Pharmaceutical Chemistry, Drug Analysis and Drug Information (FASC), Research Group Experimental Pharmacology, Center for Neurosciences (C4N), Vrije Universiteit Brussel, Brussels, Belgium; ^4^Department of Psychiatry, Vrije Universiteit Brussel, Universitair Ziekenhuis Brussel (UZBrussel), Brussels, Belgium; ^5^Department of Electrical Engineering, Eindhoven University of Technology, Eindhoven, Netherlands

**Keywords:** aHF-rTMS, DaTscan SPECT, canine model, dopamine, dopamine transporter, behavior disorder

## Abstract

**Aim:**

The neurobiological effects of repetitive transcranial magnetic stimulation are believed to run in part through the dopaminergic system. Accelerated high frequency rTMS (aHF-rTMS), a new form of stimuli delivery, is currently being tested for its usefulness in treating human and canine mental disorders. However, the short-and long-term neurobiological effects are still unclear, including the effects on the dopaminergic system. In aHF-rTMS, multiple sessions are delivered within 1 day instead of one session per day, not only to accelerate the time to response but also to increase clinical efficacy. To gain more insight into the neurobiology of aHF-rTMS, we investigated whether applying five sessions in 1 day has direct and/or delayed effects on the dopamine transporter (DAT), and on dopamine metabolites of cerebrospinal fluid (CSF) in beagles.

**Materials and methods:**

Thirteen beagles were randomly divided into two groups: five active stimulation sessions (*n* = 9), and 5 sham stimulation sessions (*n* = 4). Using DaTSCAN, DAT binding indices (BI) were obtained at baseline, after 1 day, 1 month, and 3 months post stimulation. CSF samples were collected after each scan.

**Results:**

Active aHF-rTMS significantly reduced striatal DAT BI 1 day post-active stimulation session (*p* < 0.01), and the effect lasted to 1 month (*p* < 0.01). No significant DAT BI change was found in sham group. No significant changes in dopamine metabolites of CSF were found.

**Conclusion:**

Although no significant effects on CSF dopamine metabolites were observed, five sessions of active aHF-rTMS significantly decreased striatal DAT BI after 1 day and up to 1 month post stimulation, indicating immediate and delayed effects on the brain dopaminergic system. Our findings in healthy beagles further substantiate the assumption that (a)HF-rTMS affects the brain dopaminergic system and it may pave the way to apply (a)HF-rTMS treatment in behaviorally disturbed dogs.

## 1. Introduction

Non-invasive neurostimulation techniques such as repetitive transcranial magnetic stimulation (rTMS) are widely used to treat human neuropsychiatric disorders, such as anxiety, depression, neuropathic pain, schizophrenia, drug addiction, etc. (1–4). In veterinary medicine, it has been used to treat epilepsy and anxiety in dogs (5–7). However, the underlying neurobiological mechanisms of action have not yet been completely unraveled and rTMS parameters still need to be optimized to increase clinical responses. Although daily rTMS has become the gold standard, it is thought that accelerated repetitive high-frequency TMS (multiple sessions HF-rTMS) (aHF-rTMS) not only provides faster results, but would also have a better clinical effect compared to classical daily stimulation paradigms (1, 2, 8, 9).

One neurotransmitter system in particular thought to be affected by rTMS is the dopaminergic system. This system is involved in human psychiatric and neurological disorders, such as anxiety, depression, Parkinson’s disease (PD), schizophrenia, drug addiction, attention deficit hyperactivity syndrome (ADHD), and obsessive–compulsive disorders (OCD) (10–16). In animals, it is related to stereotypic behaviors like acral lick dermatitis and compulsive checking in rats (17–19), and compulsive behavior, impulsive behavior, and anxiety related behavioral disorders in dogs (20–23). The dopamine transporter (DAT) is located on the plasma membrane of nerve terminals, transporting dopamine (DA) across the membrane. By regulating the reuptake of DA at the synaptic cleft back into presynaptic neurons, it plays a critical role in terminating DA neurotransmission and in maintaining dopamine homeostasis in the central nervous system (24–26). Thus, DAT could be used as a biomarker to investigate the dopaminergic system alteration. DAT imaging by DaTSCAN [ioflupane (123I) SPECT] has been used clinically in human medicine to detect dopaminergic deficits of striatum in psychiatric and neurological disorders, like neuro-degenerative Parkinsonian syndromes and schizophrenia with anxiety, depression, and apathy symptoms (27). In previous studies of our research group, DaTSCAN studies were conducted to determine the normal DAT BI (striatum uptake ratio) in healthy dogs (28, 29), to investigate dopaminergic dysfunction in OCD dogs (20), and to evaluate the medication treatment effect in an OCD dog (30). The latter study showed the higher DAT BI in the striatum of OCD dogs and normalization of the DAT BI after clomipramine treatment.

rTMS is thought to partly exert its action through the dopaminergic system. It has been shown that applying rTMS on prefrontal cortical areas increases striatal DA release (31–34). rTMS is a relatively new and less conventional treatment for anxiety disorders in dogs. The underlying neurobiological mechanisms of action have not yet been completely unraveled. Given the role of DA in these types of disorders in dogs, as elaborated above, it seems plausible that DA is involved in the mechanism of action of aHF-rTMS. It is not yet known whether the dopamine-releasing properties of rTMS also apply in dogs. Moreover, since dogs develop natural behavioral disorders and rely on similar neurobiological mechanisms, they may be a suitable model to evaluate the neurobiological effects of TMS.

Therefore, this study aimed to investigate the effect of 1-day active/sham aHF-rTMS on dopamine transporter, as assessed by DaTSCAN in healthy beagles. We hypothesized that active aHF-rTMS, and not sham, would alter the DAT at different time points.

## 2. Materials and methods

### 2.1. Animals

**Figure 1:**
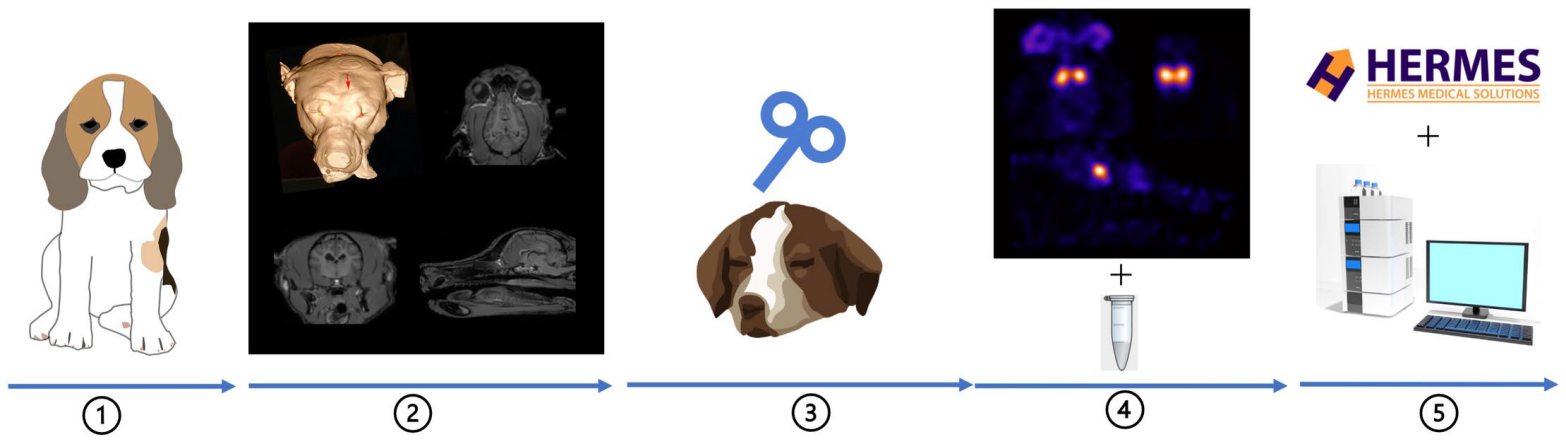
Experimental flowchart:① thirteen beagles were included, ② neuronavigation for rTMS target (red arrow), ③ aHF-rTMS sessions, ④ DaTSCAN image acquisition (transversal, sagittal and dorsal slices) + CSF sample collection, ⑤ data analysis.

Thirteen healthy beagles (8 males castrated and 5 females neutered) were included, owned by the Department of Morphology, Imaging, Orthopedics, Rehabilitation and Nutrition of Ghent University. This study (EC numbers: 2018–023) was approved by the Ghent University Ethical Committee. The guidelines for animal welfare imposed by the ethical committee were respected: the beagles were housed in groups in a 15 m^2^ kennel with wood shavings covered floor, and access to an outside area of 15 m^2^. They had access to toys filled with treats every day and were released twice a day to an enclosed playground with environmental enrichments. Moreover, the veterinary students and animal house managers walked the dogs regularly. All beagles were accustomed to the researchers and the lab environment by using positive reinforcement techniques several months before the experiment. All beagles were regularly evaluated by the veterinarians involved and caretakers. Behavior remained impeccable over the whole study period. The whole experimental flowchart is shown in Figure 1.

### 2.2. Neuronavigation

To locate the TMS target-left frontal cortex, a 3 T MRI scan was performed. Based on the high-resolution dataset, the target location was obtained by using a frameless neuronavigation system as previously described by Dockx and colleagues (35).

### 2.3. Accelerated high frequency rTMS protocol

The stimulation protocol was an exact copy of the aHF-rTMS treatment protocol used in Ghent University hospital for MDD patients (36). The 13 beagles were divided into two unequal groups: 9 beagles received 5-session active stimulation, and 4 beagles received 5-session sham stimulation.

All stimulations were performed under general anesthesia as described by Dockx et al. (37). In short, first, butorphanol was intravenously injected (0.2 mg/kg; Dolorex; Intervet Belgium NV) for sedation, then midazolam (0.2 mg/kg; Dormicum; Roche Nederland B.V.) and propofol (1 ± 2 mg/kg given to effect; Propovet Multidose; Abbott Laboratories) were applied intravenously to induce anesthesia. General anesthesia was maintained with isoflurane (1.2–1.5% to effect; Isoflo; Abbott Laboratories) in oxygen using a rebreathing system. Thereafter, the resting motor threshold (rMT) of the left motor cortex was measured. Based on the neuronavigation and MT results, a figure-of-eight coil was placed on the TMS target, namely the left frontal cortex. For the sham stimulation, the coil was placed at a 90° angle with one side contacting the skull. The aHF-rTMS parameters were set as: 20 Hz, 110% rMT for five sessions, each session contained forty 1.9 s-trains, and each train was separated by a 12 s interval.

### 2.4. Imaging procedure

Prior to the stimulation sessions, a baseline DaTSCAN (T0) scan was conducted. After the last session of rTMS, three scans were obtained: at 1 day (T1), 1 month (T2), and 3 months (T3) post stimulation. The anesthesia protocol used for the SPECT scan was as follows: the beagles were first sedated with dexmedetomidine intramuscular (375 μg/m^2^ body surface; Dexdomitor; Orion Corporation), followed by placement of an intravenous cephalic catheter. Thereafter, the DaTSCAN tracer was injected (Datscan, GE Healthcare) (138.943 ± 3.65 MBq). Three hours after the tracer injection, induction of anesthesia was started by giving propofol intravenously (1 ± 2 mg/kg given to effect; Propovet Multidose; Abbott Laboratories). After intubation, general anesthesia was maintained by isoflurane (1.2–1.5% to effect; Isoflo; Abbott Laboratories) in oxygen with a rebreathing system, equipped with capnography, pulse oximetry, and electrocardiographic monitors.

The SPECT scanner had a triple head gamma-camera (Triad, Trionix, Twinsburg, OH, United States) equipped with low energy ultrahigh-resolution parallel hole collimators (spatial system resolution, full width at half-maximum 8–9 mm) was used for all acquisitions. Data were acquired for 20 min in step-and-shoot mode (120 steps, 10 s and 3°/step) in a 128 × 128 matrix. The acquisition data were transferred to a Hermes workstation for further analysis (Hermes Gold, Version 4.18.RC.3). After each scan, CSF samples were taken under anesthesia. The dog was put in right lateral recumbency, the CSF tap was done at the cisterna magna using a 19 G needle. The CSF samples were mixed in 4:1 ratio with antioxidant (0.1 M perchloric acid (Merck, Darmstadt, Germany), 0.05% Na_2_EDTA (Sigma Aldrich, Saint Louis, United States) and 0.05% sodium metabisulfite (Merck, Darmstadt, Germany)), and stored in −80°C freezer for HPLC analysis.

### 2.5. SPECT analysis

**Figure 2:**
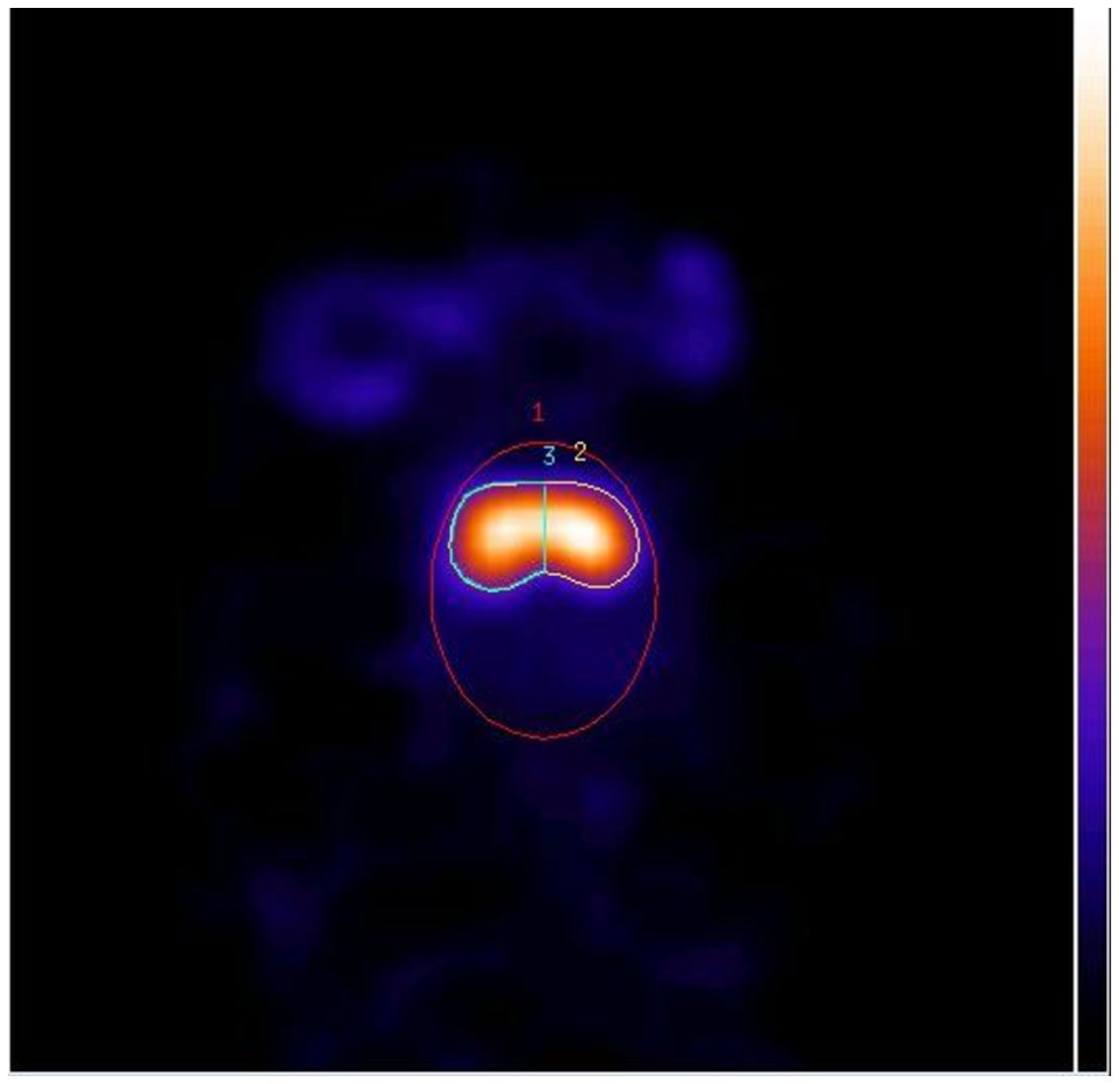
Horizontal SPECT slice of the striatum with three ROIs (1: whole brain, 2: right striatum, 3: left striatum).

Similar to our former DaTSCAN study (29), the pseudo-equilibrium and the normal DAT BI of normally behaving dogs were determined. In this study, we applied a region of interest analysis, by using a validated resolution-independent method to calculate the DAT BI in the striatum (38). In brief, several transaxial slices including the whole striatum were chosen and summed, then the left and right striatal ROI (2&3, the left and right striatum counts and pixels could be calculated) and a third elliptical ROI (1, the total brain counts and pixels could be calculated) covering the whole brain were drawn (Figure 2). For the left and right striatum uptake (DAT BI), the calculations used were as follows, and the DAT BI values were used for statistical analysis.

First, the background counts ( y1 ) was counted, the total brain counts ( c1 ) was obtained from ROI 1, the left and right striatum counts ( c3 and c2 ) were obtained from ROI 2 and 3. The formula for background counts ( y1 ) was:


(1) y1=c1−c2−c3,

the background pixels ( y2 ) was calculated based on the total brain pixels ( p1 ) and the left and right striatum pixels ( p3 and p2 ) obtained from the three ROIs:


(2) y2=p1−p2−p3,

then the background volume ( y3 ) can be calculated based on the background pixels ( y2 ) and the number of slices summed ( n ) for SPECT analysis:


(3) y3=y2∗Power(Pixelssize,3)/1000∗n,∗

for the background density ( y4 ), the formula was:


(4) y4=y1/y3,

lastly, based on the four formulas described above, the left ( y5 ) and right ( y6 ) striatum uptake (DAT BI) can be calculated as:


(5) y5=[c3−(y1∗p3/p1)]/y4,

(6) y6=[c2−(y1∗p2/p1)]/y4.

*In this study, the pixel size was 2.5 mm. The POWER is a mathematical function that computes and returns the result of a number raised to a power.

### 2.6. HPLC analysis

3,4-dihydroxyphenylacetic acid (DOPAC) and 4-hydroxy-3-methoxyphenylacetic acid (HVA) were measured in CSF samples based on previously reported methods (9, 39). Prior to the analysis, the samples were thawed and centrifuged at 15000 rpm for 15 min. The supernatant was transferred and diluted 1/2 with 0.5 M acetic acid (Fisher Scientific, United Kingdom). The samples were injected automatically on a reversed-phase liquid chromatography system (autosampler ASI-100 and HPLC pump P680 A HPG/2, Dionex, Amsterdam, The Netherlands) with electrochemical detection (potential = + 700 mV) (Amperometric Detector LC-4C, BAS, Indiana, United States). The separation was achieved using a narrowbore C18 column (XBridge, 3.5 μm 2.1x150mm, Waters, Milford, United States). The mobile phase buffer contained 0.1 M sodium acetate (Carl Roth GmbH + Co, Karlsruhe, Germany), 20 mM citric acid (Sigma Aldrich, Saint Louis, United States), 1 mM sodium octane sulfonic acid (Carl Roth GmbH + Co), 1 mM dibutylamine (Sigma Aldrich), and 0.1 mM Na_2_EDTA adjusted to pH 3.7 (mobile phase composition: 97 buffer/3 methanol [v/v]). The sample concentration was expressed as ng monoamine/100 μL.

### 2.7. Statistical analysis

DAT BI was analyzed using two-way repeated measures ANOVA. Within-subject factors were Stimulation (active or sham) and Time T0 (baseline), T1 (1 day), T2 (1 month) and T3 (3 months). All results were analyzed using the SPSS for Windows 26.0 software package. The significance level was set at *p* ≤ 0.05, two-tailed, for all analyzes. Where necessary, we applied the Greenhouse–Geisser correction to ensure the assumption of sphericity. Follow-up tests were also applied and given the small sample size non-parametric tests were performed additionally.

## 3. Results

### 3.1. SPECT data analysis

Table 1 summarizes the means, standard deviations (SDs), and standard errors of the mean (SEs) for DAT BI in each group. No hemisphere differences between the left and right striatum could be found in this study. Therefore, a generic mean for the average of the left and right hemisphere was calculated and used in the statistical analyzes.

**Table 1:** A summarization of the DAT BI results.

	Condition	*N*	Mean	SDs	SEs
Baseline (T0)	Active	9	16.53	1.47	0.49
	Sham	4	15.72	0.95	0.48
1 Day (T1)	Active	9	15.04	1.39	0.46
	Sham	4	15.39	0.84	0.42
1 Month (T2)	Active	9	13.91	1.44	0.48
	Sham	4	15.64	0.40	0.20
3 Month (T3)	Active	9	16.76	1.37	0.46
	Sham	4	15.88	0.96	0.48

**Figure 3:**
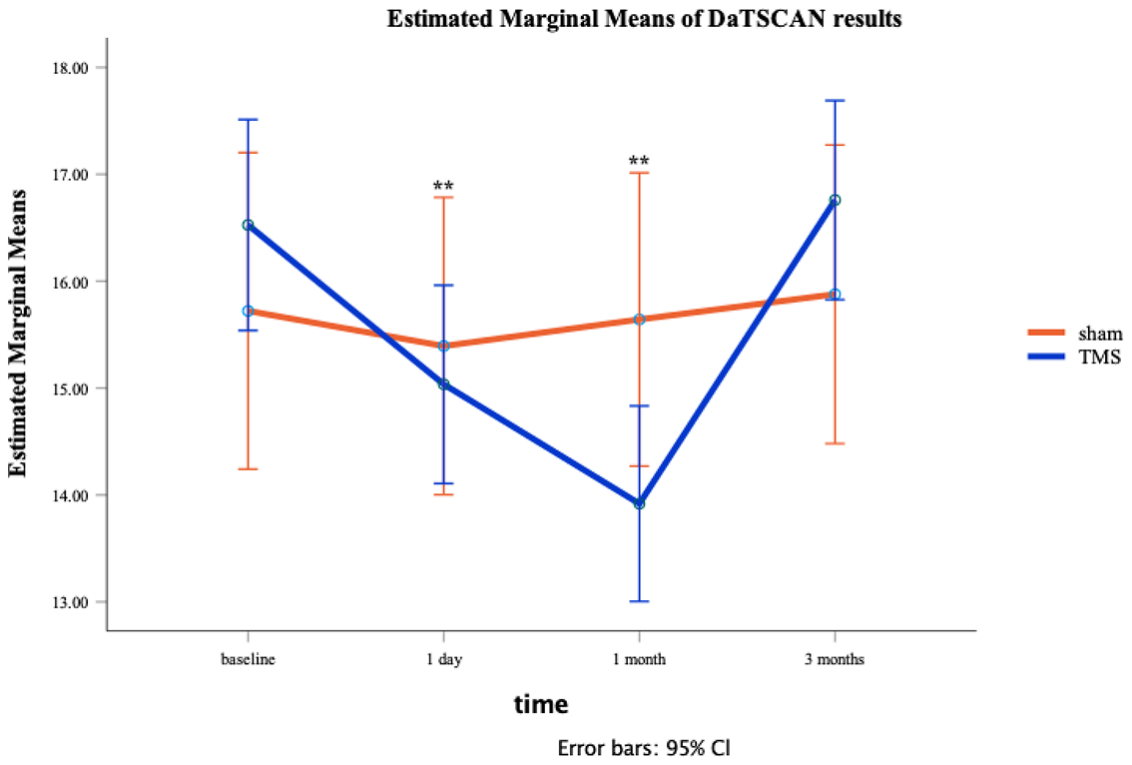
Line plot for active/sham aHF-rTMS groups at each individual time moment (T0: baseline, T1: 1 day, T2: 1 month, T3: 3 months). SEs are displayed as error bars.

In Figure 3 the estimated marginal means of DAT BI in function of the time is shown. The ANOVA showed a significant main effect for Time [*F*(3,33) = 18.97, *p* < 0.01], but not for Stimulation [*F*(1,11) = 0.02, *p* = 0.89]. Nevertheless, the crucial interaction between Time and Stimulation was significant Time [*F*(3,33) = 13.19, *p* < 0.01]. The residuals remained normally distributed. See also Figure 3.

Follow-up t-tests showed no group differences (Sham vs. Active) at T0 *t*(11) = 0.10, *p* = 0.34, T1 *t*(11) = 0.47, *p* = 0.65, and at T3 *t*(11) = 1.16, *p* = 0.22. However, DAT BI differences were observed at T2 *t*(11) = 2.30, *p* = 0.04, showing significant lower DAT BI after active aHF-rTMS [13.92 (1.44)] when compared to sham aHF-rTMS [15.64 (0.40)]. Follow-up paired t-tests also revealed that active aHF-rTMS resulted in significant DAT BI decreases after T1 (1 day) (*p* = 0.001) and T2 (1 month) (*p* = 0.001) but returned to baseline values at T3 (3 months) (*p* = 0.11). Sham aHF-rTMS showed no significant changes throughout all time measurements (*p* > 0.05), showing no impact on DAT BI (Table 1; Figure 3). Of note, given the relatively small sample size, we also performed non-parametric Friedman and Wilcoxon tests, resulting in similar findings.

### 3.2. HPLC analysis

Table 2 summarizes the means, standard deviations, and standard errors of the mean for HVA and DOPAC in CSF samples of each group. HPLC data were also analyzed by using two-way repeated measures ANOVA, and again also with non-parametric tests. No significant changes in DA metabolites DOPAC and HVA in CSF were found at any of the assessment time points (*p* > 0.05). The results can be found in the Supplementary material.

**Table 2:** A summarization of the HVA and DOPAC in CSF results (ng/100 μL).

			HVA			DOPAC		
	Condition	*N*	Mean	SDs	SEs	Mean	SDs	SEs
Baseline	Active	9	14.09	4.17	1.50	0.85	0.35	0.15
	Sham	4	13.72	2.82	1.41	0.96	0.47	0.23
1 Day	Active	9	14.79	4.48	1.50	0.96	0.44	0.15
	Sham	4	13.60	3.39	1.69	0.97	0.58	0.29
1 Month	Active	9	15.57	4.34	1.45	0.98	0.31	0.10
	Sham	4	11.22	3.35	1.68	0.58	0.32	0.16
3 Month	Active	9	13.34	3.60	1.20	1.01	0.94	0.31
	Sham	4	13.26	3.48	2.01	0.82	0.35	0.20

## 4. Discussion

This is the first report on striatal DAT BI with aHF-rTMS applied to the left frontal cortex in healthy beagles. One day of active aHF-rTMS (5 sessions/day) showed a time dependent decrease in DAT BI in the striatum immediately after 1 day and after 1 month. After 3 months, the values returned to baseline level. In the sham group, no significant changes in DAT BI could be observed at any of the given time points. The CSF dopaminergic metabolites analysis on the other hand showed no significant difference between baseline and all-time points.

Increased striatal DA release after rTMS treatment has been repeatedly reported in studies of healthy humans (31–34). Increased endogenous dopamine could provoke competition with the DaTSCAN tracer (40, 41), therefore, lead to decreased DAT BI. Excitatory projections from the prefrontal cortex (PFC) to the ventral tegmental area (VTA) play an important role in regulating the activity of VTA neurons and the extracellular levels of DA within forebrain regions in rats and primates (42). Therefore, TMS stimulus targeting the PFC may increase the activity of neurons in the PFC region, and ultimately, enhance the DA release in striatal regions (43, 44).

Involvement of the dopaminergic system in behavioral disorders in dogs such as anxiety, aggression, and impulsivity has also been reported (45–48). Lit et al. found an association between unpredictable and episodic aggression with DAT-genotype in Malinois dogs (23). Moreover, hypervigilance and a generalized state of anxiety was also reported to be associated with this DAT-genotype in these dogs, which is similar to findings in humans. A meta-analysis of human medicine elucidated the striatal DA D2 receptor decreased in patients with anxiety and obsessive compulsive disorder (49). Indeed, higher alterations in the dopaminergic system and DAT-genotype polymorphism have been associated with anxiety and post-traumatic stress disorder (PTSD) in humans, which is often accompanied with a hypervigilant state (50–52). Similar results were also found in rat studies, DAT knockout rats showed hyperlocomotion, repetitive behavior and deficits in working memory, which were relevant for OCD and ADHD (53–55). These striatal findings can be important in view of rTMS use in canine behavioral disorders. In a previous case report study, conducted by our research group, significantly higher DAT BI were found in a dog with shadow chasing compared with DAT BI in normal dog brains, in line with findings in human OCD. In this dog, DAT BI returned to normalcy after successful medical treatment (30). Similar result was found in a human study; patients with OCD after treatment with SSRIs showed a significantly decreased DAT BI in the right basal ganglia compared with baseline (56). Together, this could suggest that (aHF) rTMS treatment could be a potential therapeutic intervention for canine disorders associated with reduced DA release. Indeed, similar results were found in human psychiatric and neurological disorders. In human patients with an alcohol use disorder (AUD) study, treated with active rTMS, a reduction in DAT availability was found after 4 weeks, combined with a significant reduction in state anxiety levels, whereas the sham-treated group did not (57). Pettorruso et al. reported a decrease in DAT availability in striatal regions after 2 weeks of TMS treatment in gambling disorder (GD) (58).

However, in contrast to the brain imaging results, no significant changes of the DA metabolites DOPAC and HVA in CSF samples were found. Similarly, EI Arfani et al. also found unaltered monoamine levels in motor and depression-related brain regions after aHF-rTMS in healthy rats measured by microdialysis in subthalamic nucleus (59). No change in HVA and DOPAC was also reported in a TMS study in healthy rats measured by microdialysis in nucleus accumbens (60). It has been reported that acutely induced change in the monoamine levels in healthy rats by classical HF-rTMS returned to normal after chronic HF-rTMS measured in brain tissue homogenate, which might be the result of compensation mechanisms (61). Differences in TMS parameters such as coil shape, intensity, frequency and so on, may also regulate various aspects of DA synthesis, binding, release and reuptake differentially; the differences in sampling method may play a role as well (62). Moreover, it might be the limitation of our HPLC analysis method as only free metabolites are measured. The sulfation of HVA and DOPAC have been observed in brain, and the conjugated forms constituted 40–50% of the total amount of the metabolites in rat (63). Our antioxidant for CSF samples may cause some hydrolysis, but it is not controlled. This might contribute to the non-significant results. Uutela et al. developed a specific LC − electrospray tandem mass spectrometry method for the quantification of intact sulfate and glucuronide conjugates of serotonin, 5-hydroxyindoleacetic acid, DA, HVA, and DOPAC. They could measure low concentrations of conjugates, and the regioisomers can also be separated with their direct method (64). Measuring these conjugates could help to unravel the aHF-rTMS effects on dopaminergic system. Moreover, 3-methoxytyramine (3-MT) in the CSF could be a better biomarker of extracellular DA (65). The sample collection method may also influence the results. Advanced techniques like microdialysis and cerebral open flow microperfusion (cOFM) may do well to evaluate the accurate metabolite release in specific brain regions like striatum (66, 67). Besides, in our study, we had a very small sample size with large variation in the obtained values of the metabolites, which may also cause the non-significant results. Several additional limitations regarding this study must be considered. First, this study included healthy beagle dogs that had no diagnosis of pathological behavior, and due to our ethical restrictions, we had a relatively small sample size, which may cause the non-significant results in HPLC analysis. Secondly, during the sham treatment, an active coil was placed over the left frontal cortex, tilted 90 degrees. An active coil placed in this manner could provoke minor voltages in the underlying cortical tissue (68). Third, our protocol was performed under anesthesia, and may influence the DAT BI (69).

## 5. Conclusion

This study showed time-dependent effects of active aHF-rTMS, and not sham, resulting in decreased striatal DAT BI in healthy beagle dogs. No significant changes in dopaminergic metabolites in CSF were found. This study provided further insight into the neurobiological mechanism of aHF-rTMS in dogs, providing evidence that the dopaminergic system is involved, similar to other animal and human studies. Consequently, this similarity in neurobiological effects in dogs may potentially guide future novel treatments for behaviorally disordered dogs in veterinary medicine. In addition, such new findings on the effects of accelerated aHF-rTMS may serve as a potential animal model for human medicine. The current time-dependent brain imaging findings may also indicate that maintenance treatment could be warranted in the case aHF-rTMS treatment protocols. Further research is warranted to confirm these findings and to further explore and unravel the effects of aHF-rTMS on the dopaminergic system in psychopathological samples, in the canine as well as in the human species.

## Data availability statement

The original contributions presented in the study are included in the article/Supplementary material, further inquiries can be directed to the corresponding author.

## Ethics statement

The animal study was reviewed and approved by Ghent University Ethics Committee (EC number: 2018–23).

## Author contributions

YX: data curation, formal analysis, investigation, methodology, software, visualization, and writing–original draft. SS: formal analysis, software, visualization, and writing–original draft. KP and CB: conceptualization, project administration, resources, formal analysis, supervision, and writing–review and editing. KA and JS: conceptualization, resources, supervision, and writing–review and editing. AD: conceptualization, data analysis, and writing-review and editing. AE and DB: conceptualization, resources, formal analysis, and writing-review and editing. All authors contributed to the article and approved the submitted version.

## Funding

This study was funded by Belgium governmental FWO institution (Project number G011018N).

## Conflict of interest

The authors declare that the research was conducted in the absence of any commercial or financial relationships that could be construed as a potential conflict of interest.

## Publisher’s note

All claims expressed in this article are solely those of the authors and do not necessarily represent those of their affiliated organizations, or those of the publisher, the editors and the reviewers. Any product that may be evaluated in this article, or claim that may be made by its manufacturer, is not guaranteed or endorsed by the publisher.
