# Supplementary material for: Accelerated high frequency rTMS induces time-dependent dopaminergic alterations: a DaTSCAN brain imaging study in healthy beagle dogs
Source: Front Vet Sci. 2023 May 16;10:1154596. doi: 10.3389/fvets.2023.1154596 (PMC10228829; doi:10.3389/fvets.2023.1154596)
Supplement: Supplementary file 1 [file Data_Sheet_1.DOCX]

**Supplemental material**

**HVA results:**

For the repeated measures ANOVA test, time effect *F*(3,30)=0.04, *p*=0.99, group effect *F*(1,10)=1.06, *p*=0.33, time*effect *F*(3,30)=1.50, *p*=0.24.

For the non-parametric Friedman test, active HF-rTMS: χ2=2.33, *p*=0.51, sham: χ2=1.80, *p*=0.62; for the non-parametric Wilcoxon test, active HF-rTMS: T1 *Z*=1.01, *p*=0.32, T2 *Z*=1.25, *p*=0.21, T3 *Z*=0.89, *p*=0.37, sham: T1 *Z*=0.00, *p*=1.0, T2 *Z*=1.46, *p*=0.14, T3 *Z*=0.00, *p*=1.0.


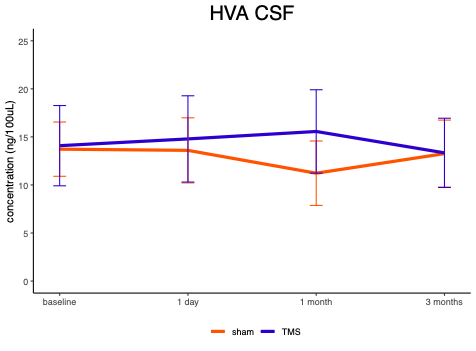


**Figure S1.** Line plot for HVA in CSF samples of active/sham aHF-rTMS groups (T0: baseline, T1:1 day, T2: 1 month, T3: 3 months). +/- 1 SDs are displayed as error bars.

**DOPAC results:**

For the repeated measures ANOVA test, main effect time *F*(3,30)=0.27, *p*=0.84, main effect group  *F*(1,10)=39.61, *p*=0.35, time*effect *F*(3,30)=0.32, *p*=0.81.

For the non-parametric Friedman test, active HF-rTMS: χ2=5.40, *p*=0.15, sham: χ2=2.60, *p*=0.46; for the non-parametric Wilcoxon test, TMS: T1 *Z*=0.89, *p*=0.37, T2 *Z*=1.13, *p*=0.26, T3 *Z*=0.53, *p*=0.59, sham: T1 *Z*=0.37, *p*=0.72, T2 *Z*=1.83, *p*=0.07, T3 *Z*=0.00, *p*=1.0.


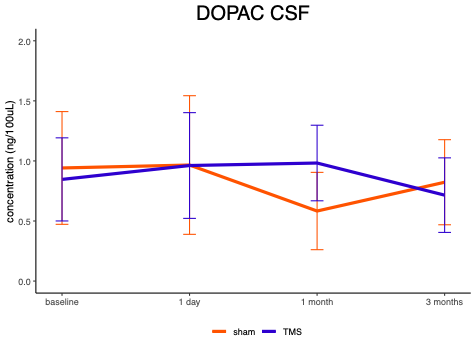


**Figure S2.** Line plot for DOPAC in CSF samples of active/sham aHF-rTMS groups (T0: baseline, T1: 1 day, T2: 1 month, T3: 3 months). +/- 1 SDs are displayed as error bars.
